# Characterization of ESBL-Producing Enterobacteria from Fruit Bats in an Unprotected Area of Makokou, Gabon

**DOI:** 10.3390/microorganisms8010138

**Published:** 2020-01-19

**Authors:** Pierre Philippe Mbehang Nguema, Richard Onanga, Guy Roger Ndong Atome, Jean Constant Obague Mbeang, Arsène Mabika Mabika, Moussa Yaro, Manon Lounnas, Yann Dumont, Zaidi Fatma Zohra, Sylvain Godreuil, François Bretagnolle

**Affiliations:** 1 Laboratoire de Microbiologie, Institut de Recherche en Ecologie Tropicale (IRET), (Centre National de recherche Scientifique et Technologique/CENAREST), Libreville B.P. 13354, Gabon; mbehangphilippe@gmail.com (P.P.M.N.); obaguejean@gmail.com (J.C.O.M.); 2 Laboratoire de Bactériologie de Recherche, Unité de recherche et d’Analyses Médicales (URAM), Centre Interdisciplinaire de Recherches Médicales de Franceville (CIRMF), Franceville B.P. 679, Gabon; raaghmabika@gmail.com; 3 UMR CNRS/uB 6282 Biogéosciences, Université de Bourgogne, 6 bd Gabriel, 21000 Dijon, France; francois.bretagnolle@u-bourgogne.fr; 4 Département de Chimie, Faculté des Sciences, Université des Sciences et Technique de Masuku (USTM), Franceville B.P. 943, Gabon; saint.claire112014@gmail.com (G.R.N.A.); lozo.10.yaro@gmail.com (M.Y.); 5 Laboratoire de Bactériologie, Centre Hospitalier Universitaire de Montpellier, Université de Montpellier, 34 295 Montpellier, France; manon.lounnas@gmail.com (M.L.); y-dumont@chu-montpellier.fr (Y.D.); s-godreuil@chu-montpellier.fr (S.G.); 6 UMR MIVEGEC IRD-CNRS-Université de Montpellier, IRD, 911 Avenue Agropolis, 34394 Montpellier, France; fatmazohrazaidi89@gmail.com; 7 Laboratoire d’Ecologie Microbienne, FSNV, Université de Bejaia, Bejaia 06000, Algerie

**Keywords:** bats, Gram-negative bacteria, ESBL, multiresistance, reservoir

## Abstract

In Gabon, terrestrial mammals of protected areas have been identified as a possible source of antibiotic-resistant bacteria. Some studies on antibiotic resistance in bats have already been carried out. The main goal of our study was to detect extended-spectrum beta-lactamases (ESBLs) that are produced by enterobacteria from bats in the Makokou region in Gabon. Sixty-eight fecal samples were obtained from 68 bats caught in the forests located 1 km from the little town of Makokou. After culture and isolation, 66 Gram-negative bacterial colonies were obtained. The double-disk diffusion test confirmed the presence of ESBLs in six (20.69%) *Escherichia coli* isolates, four (13.79%) *Klebsiella pneumoniae* isolates, and one (3.45%) *Enterobacter cloacae* isolate. The analysis based on the nucleotide sequences of the ESBL resistance genes showed that all cefotaximase-Munichs (CTX-Ms) were CTX-M-15 and that all sulfhydryl variables (SHVs) were SHV-11: 41.67% CTX-M-15-producing *E. coli*, 16.67% CTX-M-15+SHV-11-producing *E. coli*, 8.33% CTX-M-15-producing *K. pneumoniae*, 25% CTX-M-15+SHV-11-producing *K. pneumoniae*, and 8.33% CTX-M-15-produced *E. cloacae*. This study shows for the first time the presence of multiresistant ESBL-producing enterobacteria in fruit bats in Makokou.

## 1. Introduction

Bats are an important, widespread, and abundant taxon of mammals with over 1300 described species [1]. These species are considered as the reservoir of many viruses that periodically spread in human populations during disease outbreaks and whose dynamics need to be fully understood [2]. Bats have also been recognized as a potential reservoir of bacterial pathogens, although little is known concerning bat microbiota [2,3,4]. Among bats, the Pteropodidae family (large oldworld and mostly frugivorous bats) are suspected to play an important role in the dynamic of zoonosis. Many species are colonials with individuals roosting in close physical proximity that favors multiple interactions and opportunities for intraspecific pathogen transmission [4,5,6,7]. Most of the species have a wide-ranging distribution, and many urban colonies of several thousands of individuals are established in African or Asian countries, increasing the opportunities for bat–human pathogen transmission.

The existence of antibiotic multi-resistant (AMR) enterobacteria that have been recurrently documented in wildlife in recent years provide important insights into the potential role of wildlife as reservoirs of resistant bacteria. AMR enterobacteria are also pertinent markers to understand the dynamics of zoonosis and the complex transmission routes among wildlife and humans [8]. Many ways of wildlife AMR bacteria acquisition have been documented, such as contamination from human or domestic animal effluent, wastewater, and even contaminated food remains [8]. However, the possible existence of AMR enterobacteria has been poorly investigated in large fruit bats, although it could provide key information on bat–human transmission.

In several studies, antibiotic-resistant bacteria have been described in bat isolates, suggesting the possibility of these mammals being one of their environmental reservoirs [4,6,9,10,11,12,13,14,15]. In a survey of wild and captive grey-headed flying foxes (*Pteropus poliocephalus*), McDougall et al. detected the presence of resistance to several antibiotic families in 5.3% of wild flying foxes from different Australian colonies [6]. This study provided evidence of both transfer from humans to bats and from the environmental resistome to bats. A wide-ranging study that investigated the presence of antimicrobial resistance in wildlife and humans in the urban environment of Nairobi (Kenya) [16] found that fruit bats and some bird species were more likely to carry AMR *E. coli* than other taxonomic wildlife groups. This study has shown that urban environments play an important role in the dynamics of wildlife–human interactions and increase the frequency of wildlife-to-human transmission. In contrast, in a recent study, Brazilian fruit bats showed a low occurrence of resistance in their enterobacterial microbiota due to the well-preserved environment where the animal was captured [1]. However, the role of fruit bats in wildlife–human interactions is still poorly documented and needs to be more deeply investigated, particularly in the context of anthropogenic environments.

Beta-lactamases are enzymes that provide resistance to the beta-lactam family of antibiotics in Gram-negative bacteria (GNB) such as enterobacteria. These enzymes are classified into classes A–D [17]. Among these enzymes, those belonging to molecular class A extended-spectrum beta-lactamases (ESBLs) are active against expanded-spectrum cephalosporins and monobactams (Aztreonam) [18]. The introduction of extended-spectrum cephalosporin in clinical practice has caused the emergence and worldwide spreading of ESBLs in the Enterobacteriaceae such as *Klebsiella pneumoniae* and *Escherichia coli* [18,19]. The worldwide presence of EBSLs is at the origin of therapeutic failure when treating bacterial infections [19,20]. ESBL types temoneira (TEM), sulfhydryl variable (SHV), and cefotaximase-Munich (CTX-M) are the most common ESBLs found in humans, livestock, and wildlife [6]. The global spread of ESBL-producing Enterobacteriaceae, particularly CTX-M-15, creates serious therapeutic challenges [10,21,22,23,24,25,26,27,28,29,30,31,32].

In Gabon, CTX-M-15 has only been identified in ESBL-producing Enterobacteriaceae isolates from patients in the Albert Schweitzer Hospital in Lambaréné [31] and in poultry [32], but not in wildlife. Although previous studies on antibiotic resistance in Gabon have already described some phenotypes and resistance genes in wild terrestrial mammals [21,29,30], nothing is known about their dissemination in the environment. We hypothesized that bats could participate in the dissemination of antibiotic resistance in wildlife [14].

This study aimed to provide additional knowledge about the spread of ESBL-producing Enterobacteriaceae isolates from bats of the Pteropodidae family in Makokou (central Gabon).

## 2. Materials and Methods

### 2.1. Research License

The research licence for this study was obtained from the Scientific Commission on Research Authorisations of the National Centre of Scientific and Technological Research (CENAREST) (permit no. AR0033/17/MESRSFC/CENAREST/CG/CST/CSAR, dated 4 July 2017).

### 2.2. Study Area

The collection of bat fecal samples was carried out in an unprotected forest area of Makokou in Ogooué Ivindo province (located in the northeast region of Gabon) over two periods. The first capture was made at the entrance of the caves on the outskirts of the city over 10 days in May 2017. The second capture occurred near the fruit trees behind the town houses over 6 days in October 2017.

### 2.3. Collection of Fecal Samples

To capture the bats, a mist net (3000 × 2000 mm, ECOTONE^®^, France) was installed in the early evening in the narrow forests behind the human dwellings between 18:00 and 06:00. The nets were continuously monitored, and bats were immediately removed and placed in cloth bags. In the laboratory, fecal samples were collected from 68 bats. A cotton swab was rotated inside the bat rectum and was immediately discharged into 5 mL of sterile water. One millilitre of liquid sample was inoculated into BacT/Alert blood culture media (bioMérieux, Auvergne-Rhône-Alpes, France) according to a previously established protocol [30]. After incubation, enterobacteria present in the medium produced CO_2_ during their growth phase. CO_2_ product induces a decrease in pH of the culture medium. This change in pH caused the bottom of the bottle to change from blue to yellow (positive samples). The bottles containing positive samples, the bottoms of which were yellow, were kept at 4 °C pending further analysis.

### 2.4. Culture, Isolation and Identification of Colonies

In the bacteriology laboratory of the International Centre for Medical Research of Franceville (CIRMF), 50 μL of bacterial solution of each Bact/Alert vial was streaked on MacConkey agar (MCA) (bioMérieux, France) supplemented with 4 μg/mL cefotaxime and incubated at 37 °C for 24 h. After incubation, each colony, differentiated by structure and color, was picked and transferred by the same means and incubated in the same conditions. The purified colonies were subjected to biochemical identification by the VITEK^®^ 2 Compact 15 (bioMérieux, Marcy l’étoile, France).

### 2.5. Antibiotic Susceptibility Testing

ESBL production was phenotypically confirmed on Mueller–Hinton (MH) agar when the difference in the inhibition diameter zone from one of the cephalosporins (cefotaxime or ceftazidime) was alone and in combination with a disk containing clavulanic acid of ≥ 5 mm. ESBL production was confirmed by the double-disk synergy test. Antibiotic resistance was assessed by the disk-diffusion test method [33] on MH agar (BioMérieux) and the clinical breakpoints recommended by the European Committee on Antimicrobial Susceptibility Testing (EUCAST) guidelines (Version 7.1) (http://www.eucast.org/clinical_breakpoints/) using amoxicillin (25 μg), amoxicillin/clavulanic acid (20/10 μg), aztreonam (30 μg), cefepime (30 μg), cefotaxime (30 μg), cefoxitin (30 μg), ceftazidime (30 μg), cephalexin (30 μg), chloramphenicol (30 μg), colistin (50 μg), ertapenem (10 μg), fosfomycin (200 μg), gentamicin (10 μg), imipenem (10 μg), levofloxacin (5 μg), nalidixic acid (30 UI), netilmicin (10 μg), ofloxacin (5 μg), piperacillin/tazobactam (30/6 μg), piperacillin (30 μg), temocillin (30 μg), tetracycline (30 μg), ticarcillin/clavulanic acid (75/10 μg), ticarcillin (75 μg), tobramycin (10 μg), and trimethoprim/sulfonamide (1.25/23.75 μg).

The ESBL genes were identified by PCR using primers, the sequences of which are listed in Table 1 [34,35,36]. For molecular identification of ESBL genes, DNA was extracted by the boiling method, which was employed on a single colony of each isolate in a final volume of 100 μL of distilled water by incubation at 95 °C for 10 min followed by a centrifugation step [37]. The thermal cycling program consisted of initialising denaturation at 95 °C for 2 min, followed by 30 cycles of denaturation at 95 °C for 45 s. Hybridisation was carried out for 30 s (temperature was dependent on the primer (SHV: 59 °C, TEM: 63 °C, CTX-M: 57 °C)) along with extension at 72 °C for 30 s, with a final elongation step at 72 °C for 5 min. DNA from reference *blaCTX-M-*, *blaTEM*- and *blaSHV*-like-positive isolates was used as a positive control. PCR products were visualized after electrophoresis on 1.5% agarose gels containing ethidium bromide run at 100 V for 80 min. A 100 bp DNA ladder (Promega, USA) was used as a size marker. PCR products were purified using the ExoSAP-IT purification kit (GE Healthcare, Piscataway, NJ, USA) and sequenced by Sanger sequencing (first-generation sequencing) at Microsynth Seqlab AG (Göttingen Hannah-Vogt-Str.1, DE-37085 Göttingen). Nucleotide sequence alignment was done using the Mega 7 software at Microsynth Seqlab AG (Göttingen Hannah-Vogt-Str.1, DE-37085 Göttingen). Analysis and identification of these sequences were performed online using the BLAST programme available at the National Center for Biotechnology Information web page (http://www.ncbi.nlm.nih.gov).

### 2.6. Phylogenetic Analyses

Based on the examination of the relationship between the *blaCTX-M-15* and *blaSHV-11* sequences obtained by known sequences, phylogenetic trees were constructed using a set of reference sequences from GenBank. Phylogenetic analyses using ClustalW (v. 1.8.1 in BioEdit v. 7.0.9.0 software, Ibis Therapeutics, Carlsbad, CA, USA), were performed with a multiple alignment matrix of obtained partial *blaCTX-M-15* and *blaSHV-11* sequences and the GenBank reference sequences. For three constructions, we used the maximum likelihood (ML) method. The best-fitting ML model based on the Akaike information criterion was general time reversible (GTR) + Gamma + I (invariant sites). To finalize the construction, the tree was obtained by using PhyML [38,39] with nearest-neighbour interchange (NNI) + subtree pruning regrafting (SPR) branch swapping and 1000 bootstrap replicates.

### 2.7. Statistical Analyses

Data on identified bacterial species and antibiotic susceptibility testing were collected, cleaned, and entered into Statistical Package for Social Sciences (SPSS) 20.0 (SPSS Inc., Chicago, IL, USA). These data were analyzed using descriptive statistics, frequencies and bivariate analyzes (cross tables).

### 2.8. Accession Numbers

The genomes of all genes were deposited in NCBI/GenBank under the institutional numbers CH8 (2) CTX-M-15 [MK559056], CH17 (2) CTX-M-15 [MK559065], CH18 (3) CTX-M-15 [MK559057], CH38 (2) CTX-M-15 [MK559063], CH41 (1) CTX-M-15 [MK559058], CH41 (2) CTX-M-15 [MK559064], CH42 (1) CTX-M-15 [MK559059], CH42 (2) CTX-M-15 [MK559060], CH43 (1) CTX-M-15 [MK559066], CH71 (1) CTX-M-15 [MK559061], CH82 (1) CTX-M-15 [MK559062], CH38 (2) SHV-11 [MK590053], CH42 (2) SHV-11 [MK590054] and CH43 (1) SHV-11 [MK590055].

## 3. Results

### 3.1. Enterobacteria Found in Bat Faecal Samples

Over the two capture sessions, 46 *Epomops franqueti* and 22 *Megaloglossus woermanni* were caught; a total of 68 bats of the Pteropodidae family were collected. Of the 68 bacterial strains identified, 66 were Gram-negative bacteria (GNB). Among the GNB, 29 (42.65%) were enterobacteria, which consisted of 11 (37.93%) *Escherichia coli*, five (17.24%) *Klepsiella pneumoniae*, three (10.34%) *Enterobacter aerogenes*, two (5.88%) *Enterobacter cloacae*, two (6.89%) *Serratia plymuthica*, one *Citrobacter freundii* (3.45%), one (3.45%) *Enterobacter hormaechei*, one (3.45%) *Ewingella americana*, one (3.45%) *Morganella morganii*, one (3.45%) *Pantoea* sp., and one (3.45%) *Proteus vulgaris* (Table 2). The double-disk diffusion test revealed synergies that confirmed the presence of ESBLs in six (20.69%) *E. coli* isolates, four (13.79%) *K. pneumoniae* isolates, and one (3.45%) *E. cloacae* isolate (Table 2).

### 3.2. Antibiotic Susceptibility

Antibiotic susceptibility tests yielded 11 (37.93%) ESBL-producing enterobacteria. Among beta-lactams, resistance was observed by using amoxicillin (100%), ticarcillin (100%), cefotaxime (100%), ceftazidime (100%), cefpodoxime (100%), aztreonam (100%), ticarcillin/clavulanic acid (90.90%), piperacillin (90.90%), cephalexin (100%), cefepime (81.81%), amoxicillin/clavulanic acid (54.54%), cefoxitin (54.54%), ertapenem (36.36%), piperacillin/tazobactam (54.54%), and imipenem (0.00%) (Table 3).

As for other antibiotics tested on these positive ESBL strains, resistance was observed as follows: Erythromycin (100%), streptomycin (100%), ciprofloxacin (90.90%), kanamycin (81.81%), tetracycline (81.81%), trimethoprim/sulfamethoxazole (72.72%), gentamycin (63.63%), nalidixic acid (63.63%), tobramycin (63.63%), colistin (54.54%), ofloxacin (54.54%), levofloxacin (45.45%), netilmicin (36.36%), amikacin (27.27%), nitrofurantoin (18.18%), and chloramphenicol (0%) (Table 3).

Seven ESBL-producing Enterobacteriaceae from *E. franqueti* bats species and four ESBL-producing Enterobacteriae from *M. woermanni* bats species were identified. These bats belong to the family of Pteropodidae (Table 4). ESBL-producing enterobacteria generally showed resistance to amoxicillin, amoxicillin/clavulanic acid, ticarcillin, ticarcillin/clavulanic acid, piperacillin, piperacillin/tazobactam, cephalexin, cefotaxime, ceftazidime, cefpodoxime, aztreonam and cefepime (Table 4). Genetic analysis of ESBL resistance genes by PCR yielded only two types of genes: *blaCTX-M-15*, with a molecular weight of 214 bp, and *blaSHV-11*, with a molecular weight of 909 pb (Table 4).

In our findings, *E. coli* was the main enterobacterial species that was resistant to ESBLs, followed by *K. pneumoniae* and *E. cloacae*. The analysis of the nucleotide sequences resulting from the sequencing of the ESBL resistance genes showed that all *blaCTX-Ms* were *blaCTX-M-15* and all *blaSHVs* were *blaSHV-11*, according to the following distribution: 54.54% ESBL (CTX-M-15)-producing *E. coli*, 9.09% ESBL (CTX-M-15)-producing *K. pneumoniae*, 27.27% ESBL (CTX-M-15, SHV-11)-producing *K. pneumoniae* and 9.09% ESBL (CTX-M-15)-producing *E. cloacae* (Table 4).

**Figure 1:**
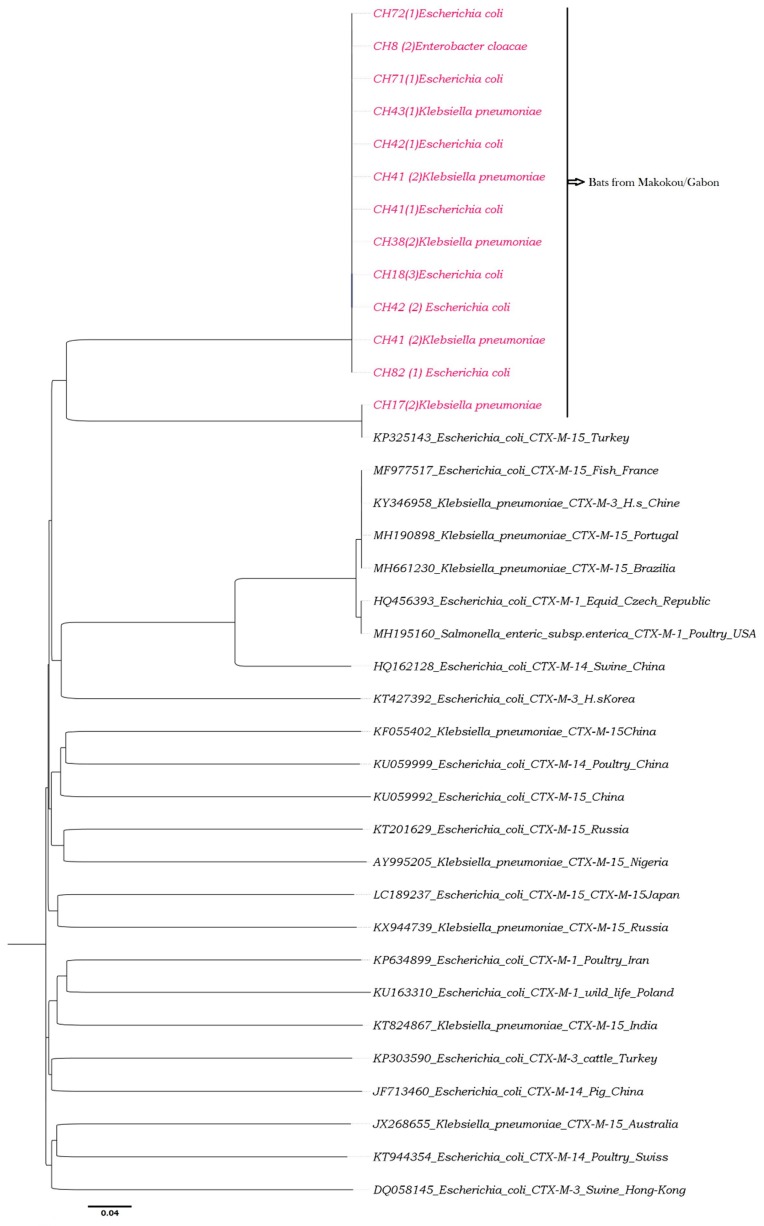
Phylogeny of CTX-M-15 from bats.

**Figure 2:**
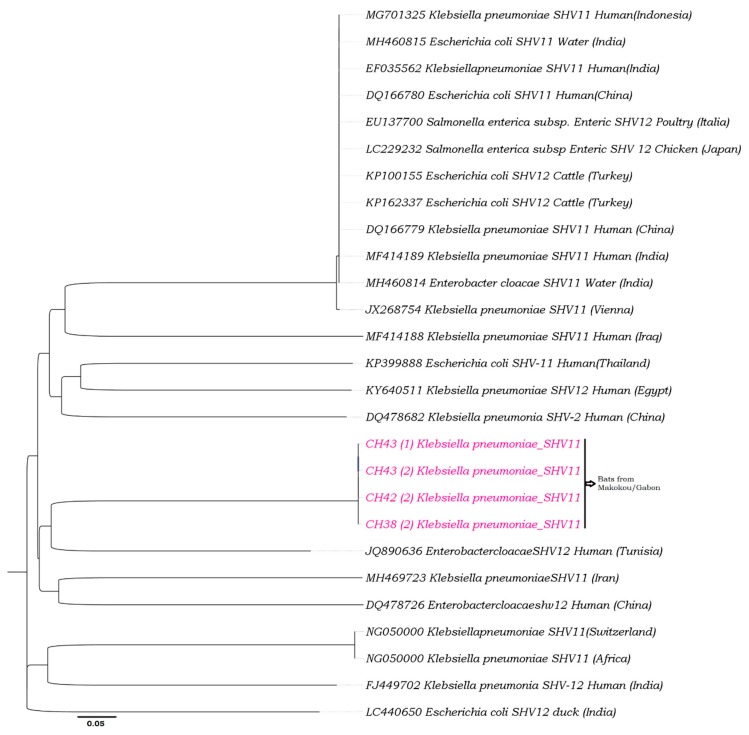
Phylogeny of SHV-11 from bats.

Phylogenetic analyses revealed that all the sequences obtained in bats that carry *blaCTX-M-15* (see Table 4) clustered with human bacterial strains carrying *blaCTX-M-15* from Turkey (Figure 1). Four sequences carrying *blaSHV-11* from *K. pneumoniae* isolates provided from bats (CH43 (1)_SHV-11, CH43 (2)_SHV-11, CH42 (2)_SHV-11 and CH38 (2)_SHV-11) clustered with human bacterial strains from Tunisia (Figure 2).

## 4. Discussion

Some studies have demonstrated that bats can carry antibiotic resistance [6,10,13,15,40,41]. Similar results were found in this study, but the *E. coli* strains were isolated from three *E. franqueti* and three *M. woermanni*, two Pteropodidae bats with different ecologies. *E. coli* is considered to be common in the physiological intestinal flora of megachiropteran bats [42,43].

The beta-lactam resistance phenotypes obtained in our study are also comparable to those obtained in a study on bats in Algeria [11], which showed resistance to amoxicillin, amoxicillin/clavulanic acid, aztreonam, cefotaxime, cefoxitin and ceftazidime. However, no ESBL resistance was found in bats in Algeria. Our study also revealed higher resistance to tetracycline (81.81%), ciprofloxacin (90.90%), and ofloxacin (54.54%) compared with that of a study carried out in Nigeria [15]. Moreover, in our data, CTX-M-15 was found either as a single resistance determinant in *E. coli* (54.54%) and *E. cloacae* and *K. pneumoniae* (9.09%) or was associated with SHV-11 in *K. pneumoniae* (27.27%). The prevalence of ESBL-producing enterobacteria (37.93%) was higher than that obtained in *E. coli* sampled in bats and other wild animals in Nigeria [13,15] and the Republic of Congo [44], in which no phenotype of ESBL resistance was found. The CTX-M-15-producing *K. pneumoniae* was found in a gorilla in its natural habitat in the Dzanga Ndoki National Park [27]. CTX-M-15 is the most highly detected genotype in human clinical settings [45,46] and in *Tadarida teniotis* (bats) in Portugal [12]. Enterobacteria producing ESBL have already been found on all continents, and the most frequently encountered is *E. coli*, followed by *K. pneumoniae* [47]. However, *blaCTX-M* beta-lactam resistance genes are the most common in the human and veterinary strains [48,49,50]. *blaCTX-M-15* is the most prevalent ESBL gene in human samples worldwide [25] and is probably the most widely distributed ESBL gene in human strains around the world [23]. In addition, *blaCTX-M-15* and *blaSHV-11* genes are recognized as plasmid-mediated resistance genes [27,51]. All of these studies show that carriage of antibiotic resistance among wildlife species may vary locally but is linked to the antibiotics used by humans [10,52,53]. 

In Gabon, the *blaCTX-M-15* genes had already been identified in ESBL-producing *Enterobacteriaceae* from patients in the Albert Schweitzer Hospital in Lambaréné and in poultry [31,32]. The presence of antibiotic resistance linked to *blaCTX-M-15* and *blaSHV-11* genes is probably linked to antibiotics used by humans, since our phylogenetic analyses show that all the sequences obtained in bats that carry CTX-M-15 or SHV-11 clustered with one human bacterial strain from Turkey and Tunisia, respectively. This suggests that the prevalence of antibiotic resistance in wild animals depends on the antibiotics consumed by human populations at each site and the density of the human population in contact with fauna [44].

In our samples, the highest prevalence of resistance was obtained in resistance to aminoglycosides (kanamycin, tobramycin and streptomycin), fluoroquinolones (ciprofloxacin and nalidixic acid) and tetracycline, which are the most common antibiotics consumed by the Gabonese people [21,54]. The congruence between the resistance prevalence and the frequency of antibiotics consumed by local people was also observed in Portugal [12] but not in Nigeria [15]. The low percentage of carbapenem resistance in our study suggests that these antibiotics are still very active against multiresistant strains [55]. Antibiotic resistance among *E. coli* isolated from wildlife may be acquired from the food [56,57,58,59] they consume, the environment [60] and water [61]; resistance may also reflect the use of antimicrobials in humans and livestock [15,52]. Further, foodborne transmission of multiresistant *E. coli* has already been described as an important source of infection in mammals [15]. Foodborne diseases such as pathogenic *E. coli* infections can result from food contamination [59]. We can therefore explain the transmission of antibiotic resistance observed in our study in three main ways. First, fruit bats may be contaminated by other mammals that already carry this type of resistance by eating the same fruits when sharing the same ecosystem [62,63]. Second, fruit bats may also be contaminated by drinking wastewater [61,64]. Most bats use open water sources for drinking water [65,66,67,68], like pools in streams, lakes, ponds, slow-flowing streams, and rivers [69,70]. These sources of water are frequently polluted, in particular near cities [61]. Several studies provide much evidence that waste water effluent and surface water are important sources of the dissemination of ESBLs in the natural environment and in particular in Africa [71,72,73]. Third, we captured some bats by hanging nets on mango branches 2 m behind human dwellings and 1 km away from the hospital. Besides these mango trees, there were other fruit trees, such as Aridan trees (*Tetrapleura tetraptera*, the fruit of which is widely consumed in Gabon) and lemon. Hence, it is possible that some fruits partly eaten by humans and contaminated have been ingested by bats. Fruit bats are potential vectors and reservoirs of pathogenic bacteria such as *E. coli*, which carry acquired resistance [2,41,59]. The risk to human health stems from the transmission of these pathogens from fruit bats to humans because fruits contaminated (half eaten or masticated fruits) by bats might be eaten by humans [2,59,74,75]. Bats can also contaminate human drinking water [61]. In this case, it would be a serious public health problem.

The phylogeny of CTX-M-15 was constructed using v. 1.8.1 in BioEdit v. 7.0.9.0 software. These analyses were performed with a multiple alignment matrix of obtained partial CTX-M-15 sequences and the GenBank reference sequences of human, poultry and swine from Africa (Nigeria), Asia (Japan, China and Hong Kong), Europe (France, Turkey, Poland, Russia, Switzerland, and Czech Republic), the Middle East (Iran), and South America (Brazil). Enterobacteriaceae carrying CTX-M-15 isolated from fruit bats of Makokou in Gabon (in pink color).

The phylogeny of SHV-11 was constructed using v. 1.8.1 in BioEdit v. 7.0.9.0 software. These analyses were performed with a multiple alignment matrix of obtained partial SHV-11 sequences and the GenBank reference sequences of human, water and cattle from Europe (Italy, Russia and Switzerland), Asia (China, Indonesia, India and Thailand), the Middle East (Iran), and Africa (Tunisia and Egypt). All sequences of SHV-11 were from *K. pneumoniae* from bats. Enterobacteriaceae carrying SHV-11 isolated from fruit bats of Makokou in Gabon (in pink color).

## 5. Conclusions

This study showed for the first time the presence of multiresistant ESBL-producing enterobacteria in Makokou fruit bats in Gabon (Central Africa). The source of the contamination has not been clearly determined, but the presence of this type of resistance in bats suggests that these wild mammals could spread ESBL-producing Enterobacteriacea over long distances or across the urban landscape.

Our results reinforce the need to monitor antimicrobial resistance in wild animals, in protected or unprotected areas, in order to assess environmental responses to anthropogenic pressures.

## Figures and Tables

**Table 1:** The different primers used for polymerase chain reaction (PCR) and bacterial identification.

Primers	Sequences	Basic Pair Length	Hybridization Temperature	References
SHV-F	5′-GATGAACGCTTTCCCATGATG-3′	214 bp	59 °C	[36]
SHV-R	5′-CGCTGTTATCGCTCATGGTAA-3′			
TEM-F	5′-AGTGCTGCCATAACCATGAGTG-3′	550 bp	63 °C	[36]
TEM-R	5′-CTGACTCCCCGTCGTGTAGATG-3′			
CTX UNIV-F	5′-TCTTCCAGAATAAGGAATCCC-3′	909 bp	57 °C	[36]
CTX UNIV-R	5′-CCGTTTCCGCTATTACAAAC-3′			

**Table 2:** Bacterial strains isolated from fruit bats.

Enterobacteria Strains	Isolates *n* = 29	ESBL Detected *n* (%)
*Citrobacter freundii*	1 (3.45)	0
*Enterobacter aerogenes*	3 (10.34)	0
*Enterobacter cloacae*	2 (6.89)	1 (3.45)
*Enterobacter hormaechei*	1 (3.45)	0
*Escherichia coli*	11 (37.93)	6 (20.69)
*Ewingella americana*	1 (3.45)	0
*Klebsiella pneumoniae*	5 (17.24)	4 (13.79)
*Morganella morganii*	1 (3.45)	0
*Pantoea* sp.	1 (3.45)	0
*Proteus vulgaris*	1 (3.45)	0
*Serratia plymuthica*	2 (6.89)	0

**Table 3:** Prevalence of antibiotic resistance in enteric bacterial strains carrying resistance for beta-lactams.

Antibiotic Agent	Number and Percentage (%) of ESBL-Producing Enterobacteria Strains by Species			
	*E. cloacae* (*n* = 1)	*E. coli* (*n* = 6)	*K. pneumoniae* (*n* = 4)	Total (*n* = 11)
Amoxicillin	1 (100)	6 (100)	4 (100)	11 (100)
Ampicillin	1 (100)	6 (100)	4 (100)	11 (100)
Amoxicillin/clavulanic acid	1 (100)	1 (16.16)	4 (100)	6 (54.54)
Ticarcillin	1 (100)	6(100)	4 (100)	11 (100)
Ticarcillin/clavulanic acid	1 (100)	5 (83.33)	4 (100)	10 (90.90)
Piperacillin	1 (100)	5 (83.33)	4 (100)	10 (90.90)
Piperacillin/tazobactam	1 (100)	0	2 (50)	3 (27.27)
Cephalexin	1 (100)	6 (100)	4 (100)	11 (100)
Cefoxitin	1 (100)	2 (33.33)	3 (75)	6 (54.54)
Cefotaxime	1 (100)	6 (100)	4 (100)	11 (100)
Cefpodoxime	1 (100)	6 (100)	4 (100)	11 (100)
Ceftazidime	1 (100)	6 (100)	4 (100)	11 (100)
Cefepime	1 (100)	4 (66.67)	4 (100)	9 (81.81)
Aztreonam	1 (100)	6 (100)	4 (100)	11 (100)
Imipenem	0	0	0	0
Ertapenem	1 (100)	2 (33.33)	1 (25)	4 (36.36)
Amikacin	1 (100)	2 (33.33)	0	3 (27.27)
Gentamycin	1 (100)	2 (33.33)	4 (80)	7 (63.63)
Kanamycin	1 (100)	4 (66.67)	4 (100)	9 (81.81)
Netilmicin	0	2 (33.33)	2 (50)	4 (36.36)
Streptomycin	1 (100)	6 (100)	4 (100)	11 (100)
Tobramycin	1 (100)	3 (50)	3 (60)	7 (63.63)
Erythromycin	1 (100)	6 (100)	4 (100)	11 (100)
Fosfomycin	1 (100)	1 (16.16)	2 (40)	4 (36.36)
Tetracycline	0	5 (83.33)	4 (100)	9 (81.81)
Colistin	1 (100)	1 (9.09)	4 (80)	6 (54.54)
Trimethoprim/sulfamethoxazole	0	4 (66.67)	4 (100)	8 (72.72)
Chloramphenicol	0	0	0	0
Nalidixic acid	1 (100)	2 (33.33)	4 (100)	7 (63.63)
Ciprofloxacin	1 (100)	5 (83.33)	4 (100)	10 (90.90)
Ofloxacin	0	3 (50)	3 (60)	6 (54.54)
Levofloxacin	0	2 (33.33)	3 (60)	5 (45.45)
Nitrofurantoin	0	0	2 (50)	2 (18.18)

**Table 4:** Antibiotic resistance profiles of Extended spectrum Beta-lactamase (ESBL)-producing Enterobacteriae from bats belonging to the family of Pteropodidae.

Colony	Species of Bat	Bacterial Strain	Profiles of ESBL-Producing *Enterobacteriae*	ESBL Gene
CH 82 (1)	*Epomops franqueti*	*E. coli*	AX-TIC-PRL-CL-CTX-CAZ-CPD-ATM-AMP-TE-STR-SXT-ERY	*blaCTX-M-15*
CH 71 (1)	*E. franqueti*	*E. coli*	AX-TIC-TIM-PRL-CL-CTX-CAZ-CPD-ATM-AMP-ERT-CIP-OFX-STR-ERY-SXT-TE	*blaCTX-M-15*
CH 41 (1)	*E. franqueti*	*E. coli*	AX-TIC-TIM-PRL-CL-FOX-CTX-CAZ-CPD-FEP-ATM-AMP-CIP-KAN-CT-E-STR-TE	*blaCTX-M-15*
CH 42 (1)	*Megaloglossus woermanni*	*E. coli*	AX-TIC-TIM-PRL-CL-CTX-CAZ-CPD-FEP-ATM-AMP-CIP-OFX-LEV-AK-CN-KAN-STR-ERY-TOB-SXT-TE	*blaCTX-M-15*
CH 18 (3)	*M. woermanni*	*E. coli*	AX-TIC-TIM-CL-FOX-CTX-CAZ-CPD-FEP-ATM-AMP-ERT-NA-CIP-AK-CN-KAN-NET-STR-E-TOB-CT-FOS	*blaCTX-M-15*
CH 41 (2)	*M. woermanni*	*E. coli*	AX-AMC-TIC-TIM-PRL-CL-CTX-CAZ-CPD-FEP-ATM-AMP-NA-CIP-OFX-LEV-KAN-NET-STR-ERY-TOB-SXT-TE	*blaCTX-M-15*
CH 8 (2)	*E. franqueti*	*E. cloacae*	AX-AMC-TIC-TIM-PRL-TPZ-CL-FOX-CTX-CAZ-CPD-FEP-ATM-ERT-NA-CIP-AK-CN-KAN-STR-TOB-CT-FOS	*blaCTX-M-15*
CH 17 (2)	*E. franqueti*	*K. pneumoniae*	AX-AMC-TIC-TIM-PRL-CTX-CAZ-CPD-FEP-ATM-CIP-OFX-KAN-CT	*blaCTX-M-15*
CH 43 (1)	*E. franqueti*	*K. pneumoniae*	AX-AMC-TIC-TIM-PRL-TPZ-CL-FOX-CTX-CAZ-CPD-FEP-ATM-CIP-CN-KAN-STR-CT-FTN-SXT-TE-	*blaCTX-M-15, blaSHV-11*
CH 42 (2)	*M. woermanni*	*K. pneumoniae*	AX-AMC-TIC-TIM-PRL-CL-FOX-CTX-CAZ-CPD-FEP-ATM-NA-CIP-CN-KAN-NET-S-TOB-CT-TE-SXT	*blaCTX-M-15, blaSHV-11*
CH 38 (2)	*E. franqueti*	*K. pneumoniae*	AX-AMC-TIC-TIM-PRL-TPZ-CL-FOX-CTX-CAZ-CPD-FEP-ATM-ERT-CIP-OFX-LEV-TOB-CN-KAN-NET-STR-FOS-TE-SXT	*blaCTX-M-15, blaSHV-11*

AX = Amoxicillin, AMC = Amoxicillin/Clavulanic Acid, TIC = Ticarcillin, TIM = Ticarcillin/Clavulanic Acid, PRL = Piperacillin, TZP = Piperacillin/Tazobactam, CL = Cephalexin, FOX = Cefoxitin, CTX = Cefotaxime, CAZ = Ceftazidime, CPD = Cefpodoxime, FEP = Cefepime, ATM = Aztreonam, ERT = Ertapenem, IMP = Imipenem, TOB = Tobramycin, KAN = Kanamycin, AK = Amikacin, NET = Netilmicin, CN = Gentamycin, NA = Nalidixic Acid, CIP = Ciprofloxacin, OFX = Ofloxacin, LEV = Levofloxacin, FOS = Fosfomycin, TE = Tetracycline, SXT = Sulfamethoxazole + Trimethoprim, C = Chloramphenicol, CT = Colistin; CTX-M = Cefotaximase-Munich; SHV = Sulfhydryl Variable, STR = Streptomycin, ERY = Erythromycin, FTN = Nitrofurantoin, CH = Chauve-souris (bats).
