# Correction: Mbehang Nguema, P.P., et al. Characterization of ESBL-Producing Enterobacteria from Fruit Bats in an Unprotected Area of Makokou, Gabon. Microorganisms 2020, *8*, 138

**DOI:** 10.3390/microorganisms8091384

**Published:** 2020-09-10

**Authors:** Pierre Philippe Mbehang Nguema, Richard Onanga, Guy Roger Ndong Atome, Jean Constant Obague Mbeang, Arsène Mabika Mabika, Moussa Yaro, Manon Lounnas, Yann Dumont, Zaidi Fatma Zohra, Sylvain Godreuil, François Bretagnolle

**Affiliations:** 1 Laboratoire de Microbiologie, Institut de Recherche en Ecologie Tropicale (IRET), (Centre National de recherche Scientifique et Technologique /CENAREST), Libreville BP 13354, Gabon; mbehangphilippe@gmail.com (P.P.M.N.); obaguejean@gmail.com (J.C.O.M.); 2 Laboratoire de Bactériologie de Recherche, Unité de recherche et d’Analyses Médicales (URAM), Centre Interdisciplinaire de Recherches Médicales de Franceville (CIRMF), Franceville BP 679, Gabon; raaghmabika@gmail.com; 3 UMR CNRS/uB 6282 Biogéosciences, Université de Bourgogne, 6 bd Gabriel, 21000 Dijon, France; francois.bretagnolle@u-bourgogne.fr; 4 Département de Chimie, Faculté des Sciences, Université des Sciences et Technique de Masuku (USTM), Franceville BP 943, Gabon; saint.claire112014@gmail.com (G.R.N.A.); lozo.10.yaro@gmail.com (M.Y.); 5 Laboratoire de Bactériologie, Centre Hospitalier Universitaire de Montpellier, Université de Montpellier, 34 295 Montpellier, France; manon.lounnas@gmail.com (M.L.); y-dumont@chu-montpellier.fr (Y.D.); s-godreuil@chu-montpellier.fr (S.G.); 6 UMR MIVEGEC IRD-CNRS-Université de Montpellier, IRD, 911 Avenue Agropolis, 34394 Montpellier, France; fatmazohrazaidi89@gmail.com; 7 Laboratoire d’Ecologie Microbienne, FSNV, Université de Bejaia, Bejaia 06000, Algeria

## Change in Abstract

The authors wish to make the following corrections to this paper [1]: the prevalences of the ESBLs genes in the abstract were not the same as those reported in the results, and as such, the authors would like to replace the original abstract.

In Gabon, terrestrial mammals of protected areas have been identified as a possible source of antibiotic-resistant bacteria. Some studies on antibiotic resistance in bats have already been carried out. The main goal of our study was to detect extended-spectrum beta-lactamases (ESBLs) that are produced by enterobacteria from bats in the Makokou region in Gabon. Sixty-eight fecal samples were obtained from 68 bats caught in the forests located 1 km from the little town of Makokou. After culture and isolation, 66 Gram-negative bacterial colonies were obtained. The double-disk diffusion test confirmed the presence of ESBLs in six (20.69%) *Escherichia coli* isolates, four (13.79%) *Klebsiella pneumoniae* isolates, and one (3.45%) *Enterobacter cloacae* isolate. The analysis based on the nucleotide sequences of the ESBL resistance genes showed that all cefotaximase-Munichs (CTX-Ms) were CTX-M-15 and that all sulfhydryl variables (SHVs) were SHV-11: 41.67% CTX-M-15-producing *E. coli*, 16.67% CTX-M-15+SHV-11-producing *E. coli*, 8.33% CTX-M-15-producing *K. pneumoniae*, 25% CTX-M-15+SHV-11-producing *K. pneumoniae,* and 8.33% CTX-M-15-produced *E. cloacae*. This study shows for the first time the presence of multiresistant ESBL-producing enterobacteria in fruit bats in Makokou.

To the correct version, as follows:

In Gabon, terrestrial mammals of protected areas have been identified as a possible source of antibiotic-resistant bacteria. Some studies on antibiotic resistance in bats have already been carried out. The main goal of our study was to detect extended-spectrum beta-lactamases (ESBLs) that are produced by enterobacteria from bats in the Makokou region in Gabon. Sixty-eight fecal samples were obtained from 68 bats caught in the forests located 1 km from the little town of Makokou. After culture and isolation, 66 Gram-negative bacterial colonies were obtained. The double-disk diffusion test confirmed the presence of ESBLs in six (20.69%) *Escherichia coli* isolates, four (13.79%) *Klebsiella pneumoniae* isolates, and one (3.45%) *Enterobacter cloacae* isolate. The analysis based on the nucleotide sequences of the ESBL resistance genes showed that all cefotaximase-Munichs (CTX-Ms) were CTX-M-15 and that all sulfhydryl variables (SHVs) were SHV-11: 54.54% ESBL (CTX-M-15)-producing *E. coli*, 9.09% ESBL (CTX-M-15)-producing *K. pneumoniae*, 27.27% ESBL (CTX-M-15, SHV-11)-producing *K. pneumoniae,* and 9.09% ESBL (CTX-M-15)-producing *E. cloacae*. This study shows for the first time the presence of multiresistant ESBL-producing enterobacteria in fruit bats in Makokou.

The authors would like to apologize for any inconvenience caused to the readers by these changes.
